# Bioactive Profiling of *Achillea millefolium* L. Growing in Kashmir: A Study on Its Phytochemical and LC‐MS Analysis, Antioxidant Properties, and Antimicrobial and Molecular Docking Analysis

**DOI:** 10.1155/cjid/9925080

**Published:** 2026-06-23

**Authors:** Zahid Ahmad Paul, Reyaz Hassan Mir, Aamir Tariq Malla, Mohd Adnan, Mitesh Patel, Reetesh Kumar, Shahid Ud Din Wani, Mohammed Iqbal Zargar, Mubashir Hussain Masoodi

**Affiliations:** ^1^ Pharmaceutical Chemistry Division, Department of Pharmaceutical Sciences, University of Kashmir, Hazratbal, Srinagar, Kashmir, 190006, India, kashmiruniversity.net; ^2^ Department of Biology, College of Science, University of Ha’il, P.O. Box 2440, Ha’il, Saudi Arabia, uoh.edu.sa; ^3^ Department of Computer Science and Bioscience, Faculty of Engineering and Technology, Marwadi University, Rajkot, Gujarat, 360003, India, marwadieducation.edu.in; ^4^ Department of Biotechnology, Galgotias University, Greater Noida, Uttar Pradesh, 201308, India, galgotiasuniversity.edu.in; ^5^ Pharmaceutics Division, Department of Pharmaceutical Sciences, School of Applied Sciences and Technology, University of Kashmir, Srinagar, 190006, India, kashmiruniversity.net; ^6^ Pharmaceutical Biotechnology Division, Department of Pharmaceutical Sciences, School of Applied Sciences and Technology, University of Kashmir, Srinagar, 190006, India, kashmiruniversity.net

**Keywords:** antioxidant, docking studies, drug discovery, pharmacology, phytoconstituents

## Abstract

**Background:**

*Achillea millefolium* L. (yarrow) is one of the oldest known medicinal plants recognized for properties, such as wound healing, fever, common cold, liver problems, nosebleeding, toothache, and headache. Yarrow, being native to Europe, grows wild in the whole of Asia, Northern Europe, and North America and also occurs wild in Kashmir.

**Objectives:**

This study aimed to investigate the antioxidant and antimicrobial properties of *Achillea millefolium* L. (AM), focusing on its ethanolic extract and its fractions (hexane and ethyl acetate).

**Methods:**

The antioxidant activity of the extracts was assessed using reducing power and DPPH radical scavenging assays. The extract and its fractions (hexane and ethyl acetate) were evaluated for antimicrobial activity by the agar well diffusion method. Additionally, LC‐MS was employed to analyze the chemical composition of the ethyl acetate fraction, and molecular docking studies were conducted to understand its mechanisms of action.

**Results:**

The ethyl acetate fraction showed the highest antioxidant activity with an IC_50_ value of 199.12 (μg/mL), antibacterial activity against *B. subtilis*, ZOI = 14.74 ± 0.16, and antifungal activity against *C. neoforman*s 34.70 ± 0.58. LC‐MS identified key compounds, including diosmin, pelargonin, and epicatechin. From the docking analysis, the highest binding affinity was monitored in the cases of diosmin with −10.7 kcal, followed by pelargonin (−9.3 kcal) and epicatechin (−8.6 kcal) with the EF‐TU complex protein.

**Conclusion:**

In summary, this study highlights the antioxidant and antimicrobial capabilities of *Achillea millefolium* L. These findings will establish the groundwork for bioassay‐guided separation of bioactive components that could serve as leads for future research to combat diseases caused by microbes that are resistant to drugs.

## 1. Introduction

Natural products derived from a wide range of sources, including plants, animals, and microorganisms, have played an essential role in the treatment of numerous ailments [1–4]. Several herbal formulations were part of traditional medical systems that were effective in treating a wide range of illnesses. These systems included those of ancient China, medieval Europe, the Americas, and India [5–10]. Worldwide, approximately 500 species of Artemisia—the dominant genus in the Asteraceae (Compositae) family—can be found in regions as diverse as Europe, Africa, Asia, Oceania, Afghanistan, Kashmir, Iran, Pakistan, Russia, India, China, and more [11–14]. The herb grows in mountainous regions of India, specifically in the states of Jammu and Kashmir, at elevations of 1500–2700 m (5000–7000 ft). Among the 45 species of the genus Artemisia found in Indian flora, *Artemisia absinthium* is considered to be one of the major species [15–18].

*Achillea millefolium* L. (AM), family Asteraceae, a highly polymorphic species with white, yellowish, or pink blooms, is a medicinal plant generally known by several names, including yarrow, nosebleed, soldiers’ woundwort, thousand leaves, or military herb [19]. The AM has been frequently employed in traditional medicine [20, 21], mostly for skin disorders, wounds, respiratory infections, and digestive issues; moreover, among other applications, for liver illness and as a moderate sedative in secondary usage [13, 22–29]. The AM has a long tradition of being applied topically to wounds, cuts, and abrasions as a potent healing herb [30]. Worldwide recognition of yarrow’s therapeutic qualities has led to its inclusion in national pharmacopeias in countries, such as Germany, the Czech Republic, France, and Switzerland [31–33]. The Indian Ayurvedic Pharmacopeia includes AM on its list of remedies for wound healing. The leaves and flowering parts of AM are used in India to treat fever and stomach issues [34, 35]. The plant has been used externally for skin inflammations, wounds, abrasions, and hepatobiliary disorders [36, 37]. AM has also been used in Kashmir traditionally for the cure of colds, fever, nosebleeding, and liver problems; locally, it is known as Pahalgam, Sultanipur, and Achilles heel [30, 38–42]. This study aimed to evaluate the phytochemical profile, antioxidant, and antimicrobial activity of the locally growing species of AM. This study focuses on examining the various extracts of AM, their phytochemical content, and antioxidant and antibacterial activities, as much research has been done on the plant’s essential oil.

## 2. Materials and Methods

The plant was collected in August 2022 at the bloom stage from the Zoohama (33.92948°N, 74.77446°E) area of the district of Budgam of Jammu and Kashmir, India. The plant was identified and authenticated taxonomically at the Herbarium of the Centre for Biodiversity & Taxonomy, Kashmir University, under specimen number 8930‐KASH.

### 2.1. Chemical Reagents Used

All of the chemicals and reagents used in this investigation are extremely pure. Chloroform, glacial acetic acid, and ferric chloride were bought from the Central Drug House (CDH). We purchased ascorbic acid, ethanol, methanol, picric acid, calcium and sodium chloride, sucrose, trichloroacetic acid, and 2,2‐diphenyl picrylhydrazyl (DPPH) from Merk. Hydrochloric acid, hydrogen peroxide, and potassium dihydrogen phosphate were acquired from Qualigens. We bought sodium hydroxide, potassium chloride, sodium dihydrogen monophosphate, ethylene diamine tetraacetate, ferric nitrate, and dimethyl sulfoxide (DMSO) from HiMedia. Ascorbic acid, ethyl acetate, and hexane were acquired from Rankem and Sisco Research Laboratories (SRL).

### 2.2. Extraction of Plant Material

Plant material was first cleaned, dried under shade, and then chopped into small pieces and coarsely powdered. An accurately weighed amount (2 kg) of AM (whole plant) was loaded into a macerator for extraction using ethanol as a solvent (6 L) as per the standard procedure [43]. The liquid was subjected to filtration followed by concentration on a rotavapor. The dried extract of AM was further subjected to fractionation (LLE) by using solvents (Merk) of different polarity, from low polarity to high polarity, according to a standard protocol [10] with slight modifications to get ethanolic extract (ETAM), hexane extract (HEAM), and ethyl acetate extract (EAAM). All plant extracts were dried completely under reduced pressure, followed by vacuum desiccation to ensure removal of residual solvent. Solvent‐only controls (using the same concentration of solvent without extract) were included in each assay to confirm that the antimicrobial effects were attributable solely to the extract components.

### 2.3. Phytochemical Evaluation

To determine the components of AM extracts, standard methods were used for the identification of phytoconstituents [10].

#### 2.3.1. Total Phenolic Content (TPC) Assessment

The method developed by Folin–Ciocalteu (FC) was used to determine the TPC. To put it briefly, 2.6 mL of distilled deionized water was mixed with 200 μL of the sample or gallic acid. A mixture was made by adding and mixing 200 μL of FC phenol reagent. After 6 minutes, 7% (w/v) Na_2_CO_3_ solution was added. The absorbance was determined at 750 nm following a 90‐min incubation period. Measured as milligrams of gallic acid equivalent (GAE) per gram of dry weight, the TPC was derived from a standard curve that utilized gallic acid as the probe. Each sample was taken three times [44–46].

#### 2.3.2. Total Flavonoid Content (TFC)

The TFC was determined using the methodology outlined by Zhishen et al. (2004). All of the extracts were combined with 120 μL of 5% sodium nitrite. Following 5 min of incubation, 120 μL of a 10% solution of aluminum chloride was introduced. After waiting 6 minutes, 800 μL of a sodium hydroxide solution with a concentration of 1 M was added to the mixture. It was discovered that the absorbance was 510 nm. Total flavonoid levels were reported as milligrams of catechin equivalents/g of dry weight (mg/g CE), with catechin serving as a probe [47].

### 2.4. In Vitro Determination of the Antioxidant Activity

#### 2.4.1. Free Radical Scavenging Assay

The capacity of AM extract to scavenge free radicals was evaluated by DPPH assay. A 0.2 mM DPPH solution in methanol was made, and 1 mL of this solution was combined with 0.5 mL of each extract (ETAM, EAAM, and HEAM) at concentrations of 100–500 μg/mL. The reaction mixture was placed in the dark for 30 min. Following incubation, the absorbance of the tubes was measured using a UV‐visible spectrophotometer at 517 nm.

#### 2.4.2. Reducing Power Assay

This assay was done by the method of Gupta et al. [48]. The standard was 1 mg/mL of ascorbic acid. Using a UV spectrophotometer, the absorbance was measured at 700 nm, and the results were also recorded in triplicate.

### 2.5. Evaluation of Antimicrobial Activity

#### 2.5.1. Agar Well Diffusion Method

The antibacterial activity of different AM extracts (ETAM, EAAM, and HEAM) involves the agar well diffusion method [49, 50]. All of the chosen bacterial colonies were first subcultured in Agar Media 2, which has a pH of 7, following a 25‐min sterilization at 121°C. To create the agar slants, 4 mL of nutrient agar medium (NAM) was added to the test tubes. The test tubes were then incubated at 37°C for an entire night. After pouring the pre‐inoculated medium into appropriately labeled and sterilized Petri plates and letting it solidify, then a sterile cork borer was used to make holes measuring 8 mm in diameter. DMSO was used as a negative control, and 100 μL of extracts at various concentrations (25, 50, and 100 mg/mL) prepared in DMSO was added to each well. Following a 30‐min standing period to facilitate the extract’s prediffusion into the medium, the plates were incubated for 16–20 h at 37°C. The inhibition zones were measured, and the results were contrasted with those of the positive control, which contained streptomycin (10 μg·mL). Three triplicates of the assay were run, and mean values were calculated. Antifungal assay [51, 52] by the agar well diffusion method was carried out in the same way as for bacterial strains, except the plates were incubated at 28°C for 16–20 h. All tests were performed in triplicate, and results were compared against both positive and negative controls to minimize the possibility of nonspecific inhibition from media interactions or impurities.

#### 2.5.2. Minimum Inhibitory Concentration (MIC)

For the determination of MIC, a broth microdilution susceptibility assay was used [53, 54]. Bacterial strains were cultured in Mueller–Hinton Agar (MHA) at 37°C overnight, while fungi were cultured in Potato Dextrose Agar (PDA) at 30°C. The ethyl acetate fraction was prepared in MHA at 48°C in a series of twofold dilutions ranging from 0.5 to 8 mg/mL for antibacterial activity and in PDA at 45°C in dilutions ranging from 0.25 to 4 mg/mL for antifungal activity. The prepared dilutions were added to the Petri plates that had been sterilized and dried at room temperature for 30 min, before spot inoculating the plates with 3  and 2 *μ*L aliquots of culture containing roughly 10^5^ and 10^3^ cfu/mL of each organism for antibacterial and antifungal activity, respectively. After 18 h of incubation at 37°C for the bacterial plates and 48 h at 28°C for the fungal plates, the MIC was determined visually. There were three replicates of each experiment run. By comparing the growth in the blank control plate to that of the plates containing different concentrations of AM ethyl acetate fraction, the inhibition of bacterial and fungal growth was assessed.

### 2.6. Liquid Chromatography–Mass Spectrometry (LC‐MS) Analysis

An effort to determine the chemical components of the ethyl acetate fraction exhibited the highest levels of antioxidant and antibacterial activity. Consequently, the Waters Alliance e2695/HPLC‐TQD was used for LC‐MS analysis on this fraction. The mass spectrometer is equipped with an LC instrument XEVO‐TQD#QCA1232 and a SUNFIRE C18 column with dimensions of 250 × 2.1 and 2.6 *μ*m. A steady column temperature of 30°C was maintained. In this experiment, the mobile phases were acetonitrile (A) and a solution of 0.1% formic acid in water (B). The flow rate was 1.5 mL min‐1. After six minutes of keeping the column at 5% A and 95% B, the concentration was gradually increased linearly: From 5% to 30% A for six to 12 minutes, from 30% to 60% A for 12 to 20 minutes, from 60% to 80% A for 20 to 26 minutes, and lastly, from 20 to 30 minutes, the concentration was maintained at 5% A and 95% BA. The spectra were recorded using both positive and negative ionization modes, spanning from m/z 150 to 2000. Mass spectra of the chemicals were compared to the online data library to identify them.

### 2.7. Docking Studies

An AutoDock Vina molecular docking study was conducted to investigate the possible mechanism of action of diosmin, pelargonin, and epicatechin against a particular protein, EF‐TU complex, in the treatment of microbial infections. The phytochemical component’s three‐dimensional (3D) structures were obtained from PubChem and converted to PDB format using Open Babel 2.4.1. The 3D protein used as a macromolecule under molecular docking analysis was retrieved from the Protein Data Bank. The EF‐TU complex protein was prepared for molecular docking by removing the ligand and water molecule [55–58]. PyMOL software was used to analyze the 3D structure in further detail [59–61]. EF‐TU was selected as the docking target due to its essential role in bacterial protein synthesis and its reported potential as an antimicrobial drug target. The three compounds were chosen based on their prior evidence of antimicrobial or bioactive potential and their representation of distinct chemical scaffolds present in the extract. Despite their structural diversity, docking was performed to investigate whether these compounds could interact with a common functional site of EF‐TU, thereby providing insights into possible shared mechanisms of antibacterial activity.

### 2.8. Statistical Analysis

Depending on the number of experiments, the data are presented as the mean ± standard deviation. We employed a standard one‐way ANOVA and Bonferroni’s multiple comparisons test to assess the data’s significance, setting a significance level at *p* < 0.05. GraphPad Prism (GraphPad Software, USA) was used for all analyses.

## 3. Results

### 3.1. Phytochemical Screening

The plant extract of AM had considerable beneficial phytochemical outcomes, as indicated by various phytochemical tests (Table 1).

**TABLE 1:** Phytochemical profile of the AM main ethanolic extract and its fractions.

S. no.	Phytochemical tests	Extract		
		Ethanolic extract	*n*‐Hexane fraction	Ethyl acetate fraction
1.	Test for carbohydrates			
	Molisch’s test	++	−	+
	Fehling’s test	++	−	+

2.	Test for alkaloids			
	Mayer’s test	+	−	−
	Dragendorff’s test	−	−	−

3.	Test for triterpenoids			
	Salkowski test	+	−	+

4.	Test for glycosides			
	Borntrager’s test	−	−	−

5.	Test for proteins			
	Ninhydrin test	−	−	+
	Biuret test	−	−	−

6.	Test for phenolic compounds			
	Ferric chloride test	−	+	++
	Lead acetate test	−	+	+

7.	Test for flavonoids			
	Shinoda test	++	−	+
	Alkaline solution test	++	+	+
	H_2_SO_4_ test	+	−	+

8.	Test for phytosterols			
	Liebermann–Burchard test	+	+++	++

9.	Test for saponins			
	Foam test	−	−	−

10.	Test for tannins			
	Ferric chloride test	−	+	+
	Lead acetate test	−	+	+

11.	Test for oils and fats			
	Copper sulfate test	+	+	+

### 3.2. TPC and TFC

**FIGURE 1:**
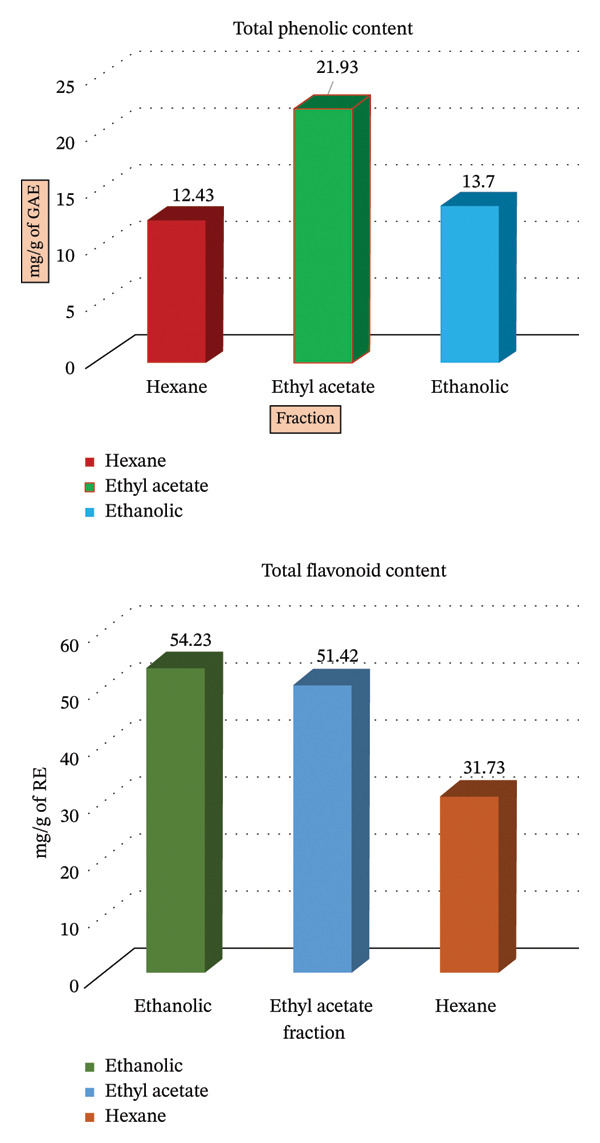
The results of TPC and TFC of AM and its various fractions.

The ethyl acetate fraction showed the presence of a maximum amount of phenolics, 21.93 mg/g of GAE, followed by an ethanolic fraction, 13.70 mg/g of GAE, and the TFC of the plant was found to be highest in the ethanolic fraction, 54.23 mg/g of GAE, followed by the ethyl acetate fraction, 51.42 mg/g of GAE. The result for TPC and TFC is summarized in Figure 1.

### 3.3. DPPH Radical Scavenging and Reducing Power Assay

**FIGURE 2:**
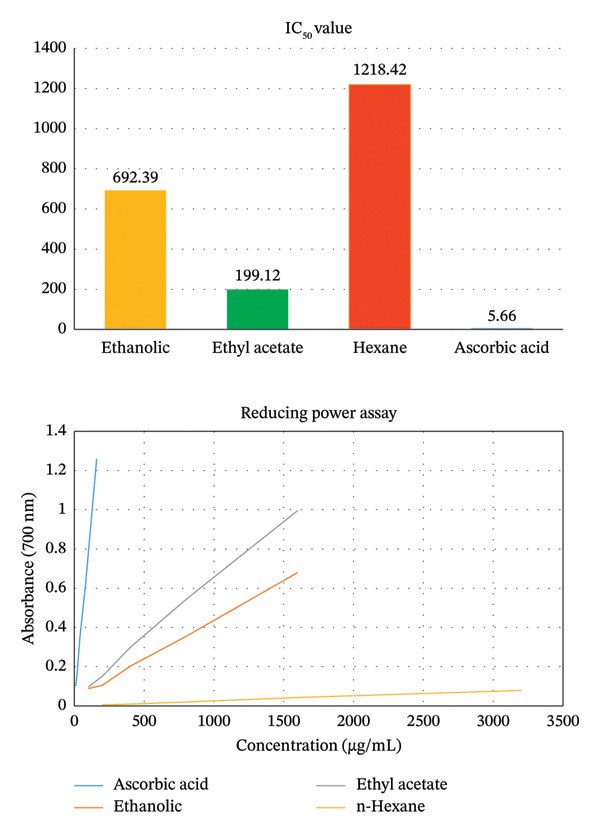
DPPH radical scavenging and reducing power assay of various fractions of AM.

The DPPH radical scavenging activity of the ethyl acetate fraction was maximum among all the extracts, with the IC_50_ values of the ethyl acetate fraction being 199.12 *μ*g/mL. The reducing ability of the AM extract and its fractions was measured by measuring the reduction of potassium ferricyanide (Fe^3+^) to potassium ferrocyanide (Fe^2+^). The study found that the reducing power of the ethyl acetate fraction was the highest among all the fractions (Figure 2).

### 3.4. LC‐MS Analysis

**FIGURE 3:**
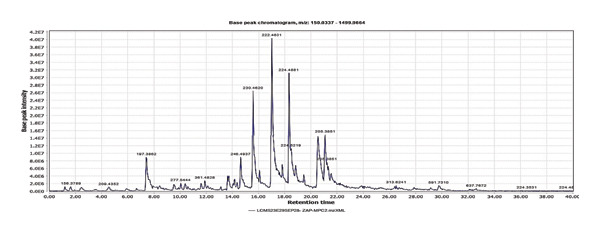
Total ion chromatogram of the ethyl acetate fraction of AM.

The ethyl acetate fraction was analyzed using LC‐MS (Figure 3). A total of 20 compounds were identified, as mentioned in the table (Table 2).

**TABLE 2:** Compounds identified by LC‐MS analysis of the ethyl acetate fraction of AM.

S. no	R. time	Compound name	Formula	Structure
1	7.42	3,4‐Dihydroxy‐L‐phenylalanine	C_9_H_11_NO_4_	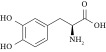
2	13.26	L‐anserine nitrate salt	C_10_H_16_N_4_O_3_	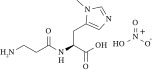
3	13.76	1,10‐Phenanthroline monohydrate	C_12_H_8_N_2_	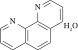
4	14.65	1‐Isothiocyanato‐9‐(methylsulfonyl)‐nonane	C_11_H_21_NOS_2_	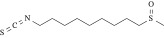
5	15.57	Melatonin	C_13_H_16_N_2_O_2_	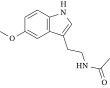
6	16.08	D‐(+)‐maltose monohydrate	C_12_H_22_O_11_	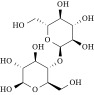
7	17.00	DL‐dihydrozeatin	C_10_H_15_ N_5_O	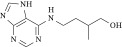
8	17.82	1‐Isothiocyanato‐8‐(methylsulfonyl)‐octane	C_10_H_19_NOS_2_	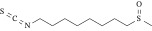
9	18.33	Methyl jasmonate	C_13_H_20_O_3_	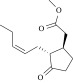
10	20.55	Pelargonin chloride	C_27_H_31_O_15_	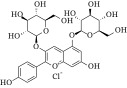
11	21.09	Etidronic acid	C_2_H_8_O_7_P_2_	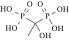
12	21.33	L‐tryptophane	C_11_H_12_N_2_O_2_	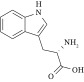
13	13.95	(+)‐Epicatechin	C_15_H_14_O_6_	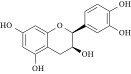
14	14.08	(+)‐Catechin hydrate	C_15_H_14_O_6_	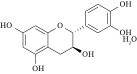
15	19.68	6‐Phosphogluconic acid barium salt hydrate	C_6_H_13_O_10_P	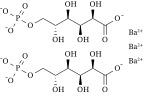
16	19.85	Gamma‐linolenic acid	C_18_H_30_O_2_	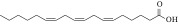
17	24.38	D‐glucosamine‐6‐phosphate sodium salt	C_6_H_14_NO_8_P	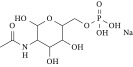
18	30.79	Xanthosine	C_10_H_12_N_4_O_6_	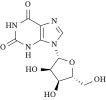

### 3.5. Antimicrobial Activity

#### 3.5.1. Agar Well Diffusion Method

**FIGURE 4:**
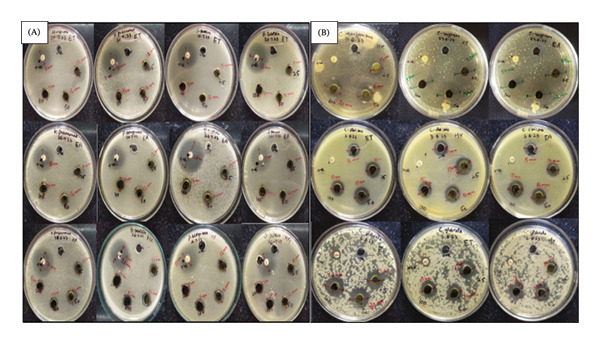
Zone of inhibition (in mm) of ethanolic, ethyl acetate, and hexane fractions of AM against bacterial strains (A) and fungal strains (B) by the agar well diffusion method.

The plant extract (AM) was evaluated for antibacterial activity by the agar well diffusion method. The ethyl acetate fraction exhibited the highest activity (ZOI = 14.74 ± 0.16) against *Bacillus subtilis*. *Staphylococcus aureus* showed the lowest activity (ZOI = 13.10 ± 0.16). The antifungal activity of the AM was found to be very significant, particularly against *Cryptococcus neoformans* (34.70 ± 0.21) and *Cryptococcus glabrata* (ZOI = 22.30 ± 0.21) (Figure 4).

#### 3.5.2. Disk Diffusion Method

**FIGURE 5:**
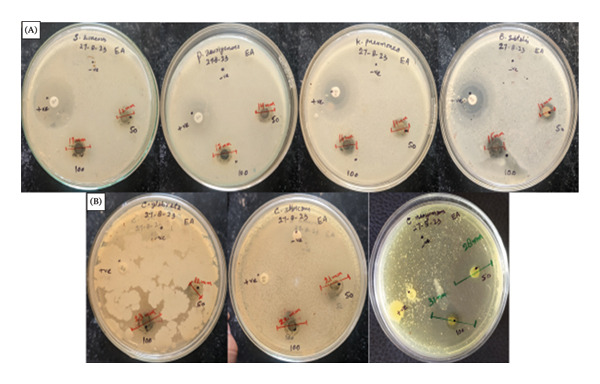
Inhibition zone (in mm) of ethyl acetate fraction against bacterial (A) and fungal (B) strains by the disk diffusion method.

The ethyl acetate fraction showed significant antimicrobial activity, so only this fraction was subjected to the disk diffusion method. The antifungal activity (Figure 5) of the ethyl acetate fraction was found to be much more significant, particularly against *C. neoformans* (34.70 ± 0.21) and *C. glabrata* (ZOI = 22.30 ± 0.21).

#### 3.5.3. MIC

**FIGURE 6:**
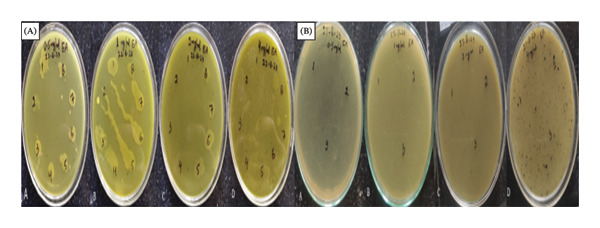
MIC values for different bacterial (A) and fungal (B) strains at varying concentrations of the EAAM.

The ethyl acetate has displayed the maximum antibacterial and antifungal activity; therefore, MIC values of only this fraction have been evaluated, and the results are shown in Figure 6.

### 3.6. Docking Studies

**FIGURE 7:**
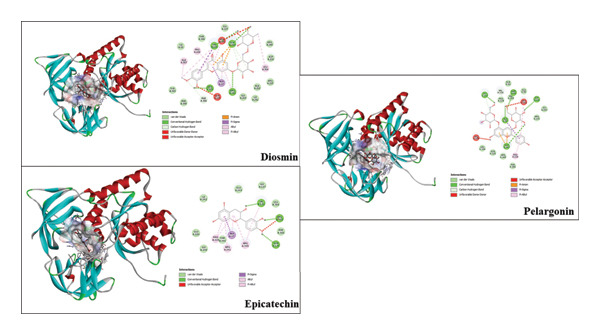
Structures of compounds diosmin, pelargonin, and epicatechin considered as drug molecules against the specific protein EF‐TU complex for the cure of microbial infections.

**FIGURE 8:**
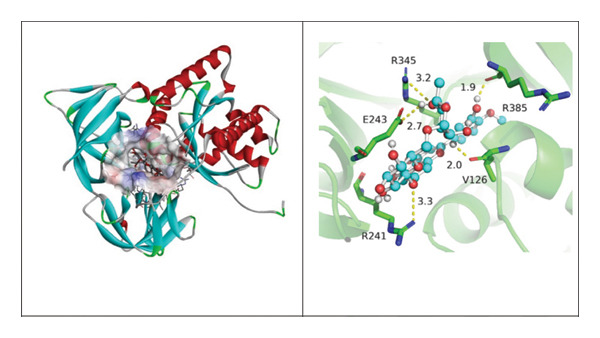
Compounds diosmin, pelargonin, and epicatechin interact with the EF‐TU complex protein. The binding affinity was monitored in the case of diosmin with −10.7 kcal.

**FIGURE 9:**
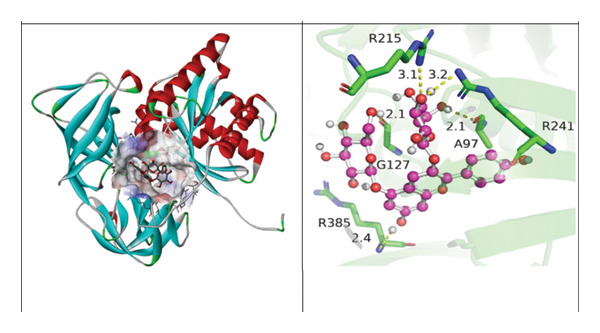
Compound pelargonin interacts with the EF‐TU complex protein. The binding affinity was monitored in the cases of pelargonin (−9.3 kcal).

**FIGURE 10:**
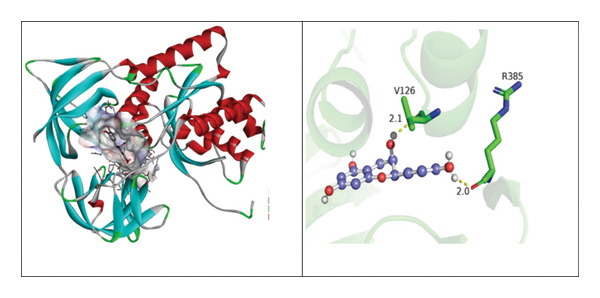
Compound epicatechin interacts with the EF‐TU complex protein. The binding affinity was monitored in the cases of epicatechin (−8.6 kcal).

Pharmacological compounds, diosmin, pelargonin, and epicatechin (Figure 7), interact with the EF‐TU complex protein. The highest binding affinity was monitored in the cases of diosmin with −10.7 kcal, followed by pelargonin (−9.3 kcal) and epicatechin (−8.6 kcal). The diosmin binds in this active site region, as shown in Figure 8. However, diosmin had an almost binding affinity with EF‐TU complex (−10.7 kcal/mol) as illustrated. The diosmin binds with V126, R241, E243, R345, and R385 amino acids of EF‐TU complex motif with 2.0‐, 3.3‐, 2.7‐, 3.9‐, and 2.0‐angstrom hydrogen bond interactions, respectively, whereas the pelargonin binds EF‐TU complex (−9.3 kcal/mol) as shown in Figure 9. The pelargonin binds with A97, G127, R215, R241, and R385 amino acids of the EF‐TU complex protein with 2.1‐, 2.1‐, 3.1‐, 3.2‐, and 2.4‐angstrom hydrogen bonds, respectively. In contrast with diosmin and pelargonin, the epicatechin bound with EF‐TU complex with −8.6 kcal/mol but had a strong interaction with V126 and R385 amino acid (Figure 10). The epicatechin binds with V126 with 2.1 angstroms and with R385 with 2.0 angstroms. The drug candidates diosmin, pelargonin, and epicatechin are shown as cyan, magenta, and blue. The hydrogen bond is shown in a yellow dotted line.

## 4. Discussion

The immense chemical variety of medicinal plants opens up boundless possibilities for the development of new plant‐based pharmaceuticals [62, 63]. In terms of primary healthcare, the World Health Organization (WHO) reports that over 80% of the global population continues to use traditional medicines derived from plants [64]. However, bioactivity investigations have only been conducted for a small fraction of medicinal plants that are used ethnopharmaceutically. For instance, just approximately 5%–10% of the approximately 250,000 species of higher plants on Earth have undergone analysis for their bioactive components [65–68]. In particular, there is a lot of interest in the potential health benefits of using innovative natural antioxidants derived from medicinal plants to protect lipids from oxidation and reduce oxidative damage [69–71]. Finding additional plant species with health‐promoting properties and investigating links between chemical composition and bioactivity are crucial in meeting the high demand for herbal remedies and providing proof of ethnobotanical uses of medicinal plants [72].

Among the extracts, the ethyl acetate fraction had the most antiradical activity (IC_50_ = 199.12 *μ*g/mL) in the DPPH scavenging assay [73]. The phytochemical examination of the Achillea species confirmed the findings of our study [74]. However, it appears that different phenolic chemicals have different concentrations depending on the species. The results presented here further confirm that polar organic solvents, such as ethyl acetate, are the best way to extract phenolic compounds, which is in line with the extracts’ potent antiradical scavenging properties. The phytochemical characterization and subsequent fractionation revealed that the appropriate ethyl acetate fractions were the only ones that contained diosmin, pelargonin, and epicatechin. Previous research has already shown a link between the levels of these phenolic compounds and enhanced antimicrobial action [75]. These four compounds are known as potent DPPH radical scavengers, and it is also possible that their presence is accompanied by an enhanced antioxidant capacity [74]. The antimicrobial activity was evaluated against two strains of Gram‐positive and two strains of Gram‐negative bacteria and three strains for antifungal activity. The ethyl acetate subextract demonstrated the highest zones of inhibition against all bacterial and fungal strains, followed by the ethanolic main extract. The ethyl acetate fraction exhibited the highest activity (ZOI = 14.74 ± 0.16) against *B. subtilis* among the bacterial strains, while *S. aureus* showed the lowest activity (ZOI = 13.10 ± 0.16). However, the positive control, which contained 10 μg·mL^−1^ of streptomycin, exhibited the highest activity (26.0 ± 0.57) against *B. subtilis* and the lowest activity (22.66 ± 0.3) against *P. aeruginosa*. The ethyl acetate fraction, at 100 mg/mL, exhibited the highest activity against *C. neoformans* (34.70 ± 0.58) and the lowest activity against *Candida albicans* (17.67 ± 0.16) among the fungal strains. However, it was discovered that amphotericin B 10 μg/mL, the positive control, was ineffective against each of these fungal strains. Notably, the 100% DMSO negative control was found to be effective against *C. albicans* (ZOI = 16.00 ± 0.57). The antibacterial and antifungal activity of the ethyl acetate fraction was also assessed using the disk diffusion method based on its highest activity among all the extracts. These outcomes validated the findings from the agar well diffusion method. The highest concentration of phenolic and flavonoid compounds, which have the potential to inhibit microorganisms, may account for the ethyl acetate fraction’s strongest antimicrobial activity [76]. Molecular docking revealed that all three compounds were able to occupy the active site of EF‐Tu with favorable binding energies. Detailed analysis of the docking poses showed that hydrogen bonding interactions were formed primarily with residues, such as Asp45, Lys56, and Thr239, which are located near the GDP/GTP binding pocket and are critical for EF‐Tu function. In addition, hydrophobic contacts with residues Val20, Ile60, and Phe229 contributed to ligand stabilization within the binding cavity. π–π stacking and van der Waals interactions were observed in the case of diosmin, pelargonin, and epicatechin, suggesting a stronger orientation and potentially higher affinity compared to the other ligands. The binding modes indicate that despite their structural diversity, the compounds share a tendency to anchor within the same functional site of EF‐Tu, exploiting a conserved interaction network. These observations provide preliminary structure–activity insights, although further molecular dynamics simulations and experimental validation will be required to confirm the biological relevance of these docking predictions.

### 4.1. Limitations

The antimicrobial activity was assessed using the agar well and disk diffusion methods, which provide only qualitative screening data. Although extracts were dried before testing, advanced analytical confirmation of complete solvent removal and purity was not performed. The molecular docking results provide predictive insights but remain speculative without supporting experimental validation. Future studies incorporating rigorous controls, advanced purification, and confirmatory in vitro or in vivo experiments will be essential to strengthen these findings.

## 5. Conclusion

Much of the work has been done on the essential oil of *Achillea millefolium*, so the current paper mainly investigates the different extracts of AM, their phytochemical content, antioxidant, and antimicrobial activity. From the Results section, it is obvious that the plant has sufficient phenolic and flavonoid content, which gives it antioxidant activity. AM has significant activity against *Klebsiella pneumoniae* and other bacterial species. Also, the plant showed maximum inhibitory action against *C. neoformans* and other fungal strains, *C. glabrata,* and *C. albicans*. LC‐MS analysis revealed the presence of important compounds, such as diosmin, pelargonin, etidronic acid, kaempferide, and epicatechin hydrate. Docking studies showed that these compounds, viz, diosmin, pelargonin, and epicatechin, interact with the EF‐TU complex protein, which is involved in the mechanism of various microbial infections. Various therapeutic and pharmacologic properties in traditional medicine are attributed to the presence of these bioactive compounds in AM. This research bridges the gap between traditional knowledge and current drug discovery, establishing the extract as a promising option for medicinal uses. In‐depth study methods and further research are required to identify more specific substances that are responsible for their pharmacological responses, and also their isolation and further development as leads for future antimicrobials.

## Author Contributions

Zahid Ahmad Paul, Reyaz Hassan Mir, and Mubashir Hussain Masoodi: conceptualization, methodology, formal analysis and investigation, and writing the original manuscript. Aamir Tariq Malla and Mohd Adnan: writing, reviewing, and editing. Shahid Ud Din Wani and Mohammed Iqbal Zargar: writing and data analysis. Mitesh Patel: writing, reviewing, and editing. Reetesh Kumar: reviewing and editing. Mohd Adnan: reviewing. Reyaz Hassan Mir: writing, editing, conceptualization, and supervision.

## Funding

No funding was received for this manuscript.

## Disclosure

All authors read and approved the final manuscript.

## Conflicts of Interest

The authors declare no conflicts of interest.

## Data Availability

The data that support the findings of this study are available from the corresponding authors upon reasonable request.
